# Atrial Fibrillation in Heart Failure: An Innocent Bystander?

**DOI:** 10.2174/157340312803760839

**Published:** 2012-11

**Authors:** MA Khan, L Neyses, MA Mamas

**Affiliations:** 1 Manchester Heart Centre, Manchester Royal Infirmary, Oxford Road, Manchester, M13 9WL, UK; 2 Manchester Academic Health Science Centre, University of Manchester, Oxford Road, Manchester, M13 9PT, UK

**Keywords:** Heart failure, atrial fibrillation, prognosis.

## Abstract

Heart failure (HF) and atrial fibrillation (AF) frequently coexist and each complicates the course of the other. The purpose of this review is to analyse the prognostic impact of AF in patients with HF and assess whether there is an advantage in targeting therapies towards the maintenance of sinus rhythm (SR) in this cohort of patients.

The presence of AF in patients with HF has been reported to be independently associated with an increase in mortality in many studies and this increased risk is observed in those with both preserved and impaired LV systolic function. The optimal strategy for targeting AF in patients with HF is unclear but recent randomised controlled studies indicate no significant prognostic advantage associated with a rhythm control strategy as compared to a rate control strategy. A number of small studies have investigated the role of both cardiac resynchronization therapy (CRT) and AF catheter ablation for the maintenance of / conversion to SR in patients with HF with initial promising results although larger randomised controlled studies will need to be performed to define the role of these modalities in the treatment of this cohort and whether preliminary benefits observed in these studies translate to improvements in longer term prognosis. Finally, there has been a focus on modifying the arrythmogenic atrial substrate and neurohormonal milieu by pharmacological means in order to prevent AF although it remains to be seen whether this approach proves to be efficacious with improvements in clinically relevant outcomes.

## INTRODUCTION

Heart failure (HF) and atrial fibrillation (AF) are amongst the commonest cardiovascular conditions encountered in clinical practice and frequently coexist. Heart failure predicts the development of AF and conversely the presence of AF predicts the development of HF [1]. Heart failure prevalence has reached the proportions of a global epidemic with an estimated prevalence of 3-20 cases /1000 population rising to above 100 cases /1000 population in those aged over 65 years [2]. Similarly, the annual incidence of heart failure in middle aged men and women is 0.1-0.2 % rising steadily to 2-3 % in those aged above 85 years [2]. Extrapolating from available evidence, as many as 30 million people in Europe may have heart failure [3]. National Health and Nutrition Examination Survey (NHANES) data from 2005 to 2008 indicates that the prevalence of heart failure in Americans (over 20 years of age) is around 5.7 million. The lifetime likelihood of developing heart failure has been estimated as 1 in 5 and this risk rises with an ageing population. HF incidence approaches 10 per 1000 in above 65 year old group [4]. 

Atrial fibrillation is the most common sustained arrhythmia seen in clinical practice [5]. The Framingham as well as the Rotterdam studies estimate around 25% lifetime risk of developing AF. The prevalence of AF is estimated between 2.7 to 6.1 million in the United States. This is expected to rise to between 5.6 and 12 million [4]. The AnTicoagulation and Risk Factors In Atrial Fibrillation (ATRIA) study predicted that the prevalence will rise 2.5 times by 2050 [6]. The incidence rises steeply with age, rising to 17.4% in those above 85 years of age [7]. Similar to HF, AF also carries an enormous burden of morbidity, mortality and healthcare costs [8]. 

AF and HF frequently co-exist. The EuroHeart survey studied hospital admissions for heart failure in 24 European countries over a 6-week period in 10,701 patients and demonstrated that 34 % of patients had previous AF while 9% had new onset AF [9]. Whilst AF and HF frequently co-exist, it remains unclear as to whether the presence of chronic AF has a prognostic impact on outcomes in patients with HF. The purpose of this review is therefore to analyse the prognostic impact of AF in patients with HF and to assess whether there is an advantage in targeting therapies towards the maintenance of sinus rhythm in this cohort of patients. 

## EPIDEMIOLOGY

HF and AF share many common risk factors and frequently coexist. For instance up to 20% with AF have HF and 5-50% with HF suffer with AF as well [1]. Factors such as hypertension, coronary atherosclerosis, diabetes mellitus, obesity and structural heart disease (ischaemic, nonischaemic, valvular) all predispose to HF as well as AF. Both increase exponentially with increasing age. Moreover, AF becomes more prevalent with worsening severity of HF. Thus it can range from less than 10% in those with NYHA class 1 symptoms to 50 % in those in NYHA class 4 [10]. According to the Acute Decompensated Heart Failure National Registry (ADHERE) around 30% of patients hospitalized for decompensated HF have AF [11] whilst the ALPHA study showed around 20-30% of NYHA class 2-3 patients had AF [12]. This compares to 50% patients with NYHA class 4 symptoms in the Cooperative North Scandinavian Enalapril Survival Study (CONSENSUS) who had concomitant AF [10]. 

## INTERPLAY BETWEEN AF AND HF

There is a complex inter-relationship between AF and HF. Each adversely affects and complicates the course of the other [13]. HF provides a substrate for the development of AF through a number of mechanisms such as atrial dilatation, fibrosis and electromechanical remodelling [13]. Neurohormonal activation and dysregulation of intracellular calcium may also play a role [14]. Similarly, AF predisposes to HF through a variety of mechanisms including tachycardia–related cardiomyopathy, loss of atrial kick, reducing ventricular diastolic filling time and functional mitral/tricuspid regurgitation [1]. The development of AF in HF may contribute to decompensation. For example, irregularity of the RR interval as seen in AF may affect haemodynamics adversely independent of the heart rate [15]. Pozolli *et al. *studied the haemodynamic effects of new onset AF in heart failure patients. 344 patients with heart failure and sinus rhythm at baseline were prospectively followed up for the onset of AF. They showed that the onset of AF led to significant reduction in cardiac index, increased bi-atrial dimensions and functional atrioventricular valve regurgitation. This coincided with a decline in NYHA class as well as peak exercise oxygen consumption [16]. The pathophysiology of AF in CHF has previously been reviewed by Lubitz *et al. *and is beyond the scope of this review [8].

## PROGNOSTIC IMPACT OF AF IN HF PATIENTS

A number of studies in patients with LV systolic dysfunction have shown that AF has an impact on prognosis. Retrospective analysis of the Studies Of Left Ventricular Dysfunction (SOLVD) by Dries *et al. *(involving 6517 patients with LVEF of less than 35%) showed that AF was associated with an increased risk for all-cause mortality in comparison to those in sinus rhythm (34% vs. 23%). This was applicable to asymptomatic as well as symptomatic patients and mainly attributable to an increased risk of pump failure deaths [17]. Similarly, the Candesartan in Heart FailureAssessment of Re duction in Mortality and Morbidity (CHARM) investigators showed an increased and independent effect of AF on cardiovascular outcomes in patients with either reduced or preserved LV systolic function [18]. Both the above retrospective analyses are, however, limited by the fact that data for these studies were derived from subgroup analysis. Prospective data from Stevenson *et al. *looked at the influence of AF on all-cause mortality in 390 patients with advanced systolic heart failure. They concluded that AF is an independent predictor of all-cause mortality (actuarial survival at 1 yr with AF 52% vs. 71% with sinus rhythm). Interestingly, atrial fibrillation was associated with increased 1-year mortality only in patients with a pulmonary capillary wedge pressure lower than 16mmHg rather than the ones with higher filling pressures [19] suggesting that the relative risk of sudden death is highest in patients with relatively better ventricular function as compared to patients with more advanced ventricular dysfunction.

An adjusted meta-analysis of 16 studies (7 randomised trials and 9 observational studies) involving 53969 patients by Mamas *et al. *suggested worse prognosis with AF irrespective of systolic function [20]. They showed that AF has a deleterious effect on total mortality with an odds ratio of 1.40 (95% CI 1.32-1.48, P<0.0001) in randomised trials and an OR of 1.14 (95% CI 1.03-1.26, P<0.05) in observational studies. Ahmed *et al. *retrospectively studied an older population (mean age of 79 years) who had heart failure as the primary discharge diagnosis (no distinction was made between systolic and diastolic LV dysfunction). 4-year mortality rates and 30-day readmission rates were analysed. Multivariate analysis revealed a significant 52% increased risk of 4-year mortality but insignificant higher risk of readmission at 30 days [21]. 

Left ventricular diastolic dysfunction may also play an important role in patients with atrial fibrillation [1]. Not only does it predict the development of AF in HF but has also been connected with increased mortality [22]. Results from the CHARM study showed that although the absolute risk for all-cause mortality was highest in the low ejection fraction cohort, the relative increase in risk was highest in heart failure with preserved left ventricular ejection fraction cohort (HR 1.37, 95% CI 1.06 to 1.79) as compared to that with reduced ejection fraction (HR 1.22, 95% CI 1.04 to 1.43)[18].

There are a limited number of studies that assess *new onset acute AF* in patients with heart failure as an independent prognostic factor in comparison to those with chronic or paroxysmal AF. Borleffs *et al. *collected data on a prospective basis in patients receiving ICD implants. Those with chronic AF demonstrated twice the mortality as well as device discharge (both appropriate and inappropriate) than those in sinus rhythm. Paroxysmal or persistent AF, on the other hand, did not have increased mortality but thrice the inappropriate shocks [23]. Caldwell *et al. *interrogated ICDs of patients with severe heart failure. As many as 27% of patients previously thought to be in sinus rhythm were found to have silent episodes of paroxysmal AF. There was a trend towards increased mortality but not on thromboembolic events or hospital admissions [24]. Chamberlain *et al. *showed through their community study that there is a significant excess risk of all-cause mortality in patients with AF in HF as compared to those with HF without AF. Compared to patients in sinus rhythm, those with AF prior to HF had a 29% increased risk of death, while those who developed AF after HF had more than a 2-fold increased risk of death [25]. The EuroHeart Failure survey showed that the increase in mortality with acute new onset of AF was higher than that in chronic AF (12% vs. 7%). This was possibly associated with more haemodynamic compromise with faster heart rates as well as a higher use of anti-arrhythmic agents as compared to those known to have AF in the past [9]. 

A number of studies have shown worse prognosis in HF patients with an ischaemic aetiology. The Valsartan in Acute Myocardial Infarction (VALIANT) study dealt with post myocardial infarction systolic impairment and the potential effect of previously known or new-onset AF on mortality in such patients. Mortality was increased at 3 years in both AF groups (those known to have chronic AF at baseline as well as those who developed new AF concomitantly with the myocardial infarction) [26]. Raunso *et al. *followed up participants of the Echocardiographic Heart of England Screening (ECHOES) study. A total of 2881 patients were followed up for 4 years. AF showed increased mortality risk only in patients with coronary artery disease (CAD) whilst it had no prognostic influence in those patients without an ischemic substrate [27]. Analysis of the Danish Investigations of Arrhythmia and Mortality on Dofetilide in Congestive Heart Failure (DIAMOND-HF) data had shown similar results in comparing ischaemic versus non ischaemic subsets [28]. When 3587 HF patients with and without ischaemic heart disease were followed for up to 8 years, there was a significant impact of AF on mortality in those with ischaemic heart disease [HR of 1.25 (95% CI: 1.09–1.42) and P < 0.001] as compared to those without ischaemic heart disease [HR of 1.01 (95% CI: 0.88–1.16) and P = 0.88]. It has been shown that AF is associated with reduced myocardial blood flow and increased coronary vascular resistance [29]. It is possible that presence of AF thus adds further ischaemic burden in such patients leading to a worse prognosis.

In contrast, other studies have shown no independent prognostic effect of AF. An analysis of the Carvedilol or Metoprolol European Trial (COMET) cohort by Svedberg *et al. *failed to demonstrate an independent prognostic influence when adjusted for other predictors of prognosis [30]. However one of the major limitations of this study was that the presence of AF was probably underestimated given that the criteria for diagnosing AF was limited to a single baseline ECG thus potentially missing future development of AF during the course of the study or the presence of paroxysmal AF. Correll *et al. *demonstrated that AF at baseline was associated with increased all-cause mortality and all-cause mortality/hospitalization. However, when adjusted for baseline covariates, it lost its independent prognostic impact on all-cause mortality and only retained it for the combined end point of all-cause mortality/hospitalization [31]. Again this may have missed the impact of paroxysmal AF. The study is underpowered due to small numbers and can only be regarded as hypothesis generating for new onset AF and its influence on long-term outcomes. Mahoney *et al. *showed that in patients with advanced heart failure referred for cardiac transplantation, AF was not associated with a reduced event free survival [32]. Instead, the prognosis for heart transplant population depends on the baseline resting heart rate irrespective of the presence of AF or sinus rhythm [33]. The severity and end-stage nature of heart failure in this cohort of patients, as well as the cross-sectional design of the study and small number of patients limits the applicability of these observations to a general HF population. Similarly, retrospective analysis of the Vasodilator-Heart Failure Trials (V-HEFT) in mild to moderate HF did not show AF as independent predictor of mortality [34]. Patients in AF in the study had higher LVEF than the ones in sinus rhythm and may have influenced the outcome. Japanese registry data for hospitalized patients showed that although AF is common in hospitalized patients, it did not influence long term outcomes independently [35]. This retrospective study did not cater for the impact of subsequent or recurrent AF and may have underestimated the prognostic effect. Prospective data from the Heart Failure Survey in Israel (HFSIS) showed an increased crude mortality rate in hospitalized patients both during index admission as well as at 1 and 4 years follow up. The survey, however, failed to prove an independent effect. The prognostic effect was largely explained by comorbidities [36]. Similarly, meta-analysis of 20 studies including 9 RCTs and 11 observational studies representing 32946 patients by Wasywich *et al. *demonstrated worse outcomes in those with AF when compared to sinus rhythm. It is unclear whether this was an independent effect or due to other prognostic variables such as age, comorbidities and HF severity [37]. The adjusted meta-analysis by Mamas *et al. *[20] has shown that the prognostic effect of AF on mortality persisted after multivariate adjustment. 

## EFFECT OF VARIOUS THERAPEUTIC MODALITIES ON PROGNOSIS

Whilst many of the studies outlined above have shown that the presence of AF in patients with HF is associated with an adverse prognosis, it remains unclear whether targeting AF with a view to maintaining sinus rhythm improves outcomes. AF management has received a great amount of interest over the years and a variety of therapeutic options have been developed to both optimise rate control and promote cardioversion / maintenance of sinus rhythm. 

Pharmacological agents have long been the mainstay of AF management in HF. A number of trials have shown the benefit of ventricular *rate control* with beta-blockers and digoxin (as adjunct). A post-hoc retrospective analysis of the US Carvedilol HF trial was carried out looking specifically at a subgroup of patients who had AF at baseline. It not only demonstrated improved LV ejection fractions in the carvedilol group (as compared to placebo) but there was also a trend towards reduced combined end point of death/hospitalization [38]. The analysis of the same subgroup also showed similar survival in patients on carvedilol who were also on digoxin suggesting an added effect of the latter. Studies had already shown reduced hospitalization and improved symptom control with digoxin therapy [39]. Sub group analysis of the 600 AF patients in the COMET trial cohort showed a similar survival benefit with carvedilol [30]. The Digitalis Investigation Group (DIG) trial showed that although digoxin did not affect mortality in HF yet it reduced the number of hospitalizations. Ahmed *et al. *conducted a pre-specified 2-year post hoc analysis of DIG trial data specifically looking at patients with AF and HF. They showed a significant reduction in mortality as compared to placebo (27% vs. 33%) when higher risk heart failure patients with a LVEF of less than 25 % and AF were considered [40]. 

The influence of pharmacological *rhythm control* of AF in HF has mainly been studied using amiodarone and dofetelide. Amiodarone is effective in maintaining sinus rhythm and has a neutral effect on survival in moderate/severe systolic dysfunction as shown in the Survival Trial of Antiarrhythmic Therapy in Congestive Heart Failure (CHF-STAT) and AF-CHF trials [41,42] but is associated with a significant long-term risk of adverse effects. 79% of patients prescribed dofetilide in DIAMOND trial remained in sinus rhythm. It reduced hospital admissions with heart failure [43]. The trial mainly looked at safety/efficacy in ischaemic systolic heart failure. However, there remains a risk of torsade de pointes (1.6%) even when initiated in hospital with close monitoring [43]. Dofetelide, however, is not available in Europe. Dronedarone is an iodine-free amiodarone derivative which has shown a promising adverse effect profile. ATHENA was a placebo-controlled, double blind study comparing dronedarone with placebo in atrial fibrillation. A post hoc analysis (in patients with stable HF with LVEF less than 40% and NYHA II/III symptoms) demonstrated reduced cardiovascular events (first cardiovascular hospitalization or death from any cause) [44]. However, ANDROMEDA trial which studied the anti-arrhythmic dronedarone in patients with advanced heart failure (LVEF<35%) who had been recently hospitalized with new or worsening heart failure had to be terminated prematurely because dronedarone increased mortality in patients with severe heart failure. [45].

The debate of* a Rate vs. Rhythm control* *strategy* in patients with / without HF is controversial. A number of trials indicate no added benefit in terms of long term outcomes from rhythm control in comparison to rate control. These include the Rate Control versus Electrical Cardioversion for Persistent Atrial Fibrillation (RACE)[46], the Atrial Fibrillation Follow-Up Investigation of Rhythm Management (AFFIRM)[47], the How to Treat Chronic Atrial Fibrillation (HOT CAFÉ)[48], the Strategies of Treatment of Atrial Fibrillation (STAF)[49] and Pharmacological Intervention in Atrial Fibrillation (PIAF)[50]. These trials, however, were not exclusive to HF and the results may not necessarily be applicable to patients with heart failure. AF-CHF was a prospective trial in the HF population. 1376 patients with systolic heart failure were randomized to amiodarone or rate control respectively and followed up for 3 years for mortality, heart failure hospitalization and stroke [42]. It also arrived at similar results-there was no significant difference in death from cardiovascular cause in rhythm control patients as compared to rate control (27% vs. 25%). Secondary outcomes including death from any cause, stroke, worsening heart failure and composite of death from cardiovascular cause, stroke and worsening heart failure did not reveal any difference as well. However, the proportion of patients in the rhythm control arm who were truly free of AF was 80% (possibly 65% looking at the overall 3 year follow up visits as well as the 21% who crossed over to the rate control arm due to inability in maintaining sinus rhythm) [14]. A recent meta-analysis by Caldeira *et al. *analysed the 4 main RCTs of AF rate vs. rhythm control in heart failure incorporating 2486 patients. There was no significant difference in terms of mortality and stroke [51]. Registry on Cardiac Rhythm Disorders Assessing the Control of Atrial Fibrillation (RECORD-AF) was a community based prospective, multinational observational study that analysed data from 5171 patients of AF. It looked at real life experience in unselected patients with AF including those with and without HF [52]. It showed that outcomes in AF were not related to either a rate or rhythm control strategy. Conversely, Guglin *et al. *presented a post hoc analysis of the AFFIRM cohort looking at the rhythm control arm. Sinus rhythm was associated with fewer symptoms of HF (assessed by NYHA functional class) and improved functional status (assessed by 6-minute walk test) [53]. Also CHF-STAT subgroup analysis by Deedwania *et al. *showed that amiodarone therapy was more effective in converting AF to sinus rhythm as compared to placebo (31% vs. 8%). It not only prevented new onset AF throughout the course of the study but was also effective in maintaining a lower ventricular rate in those who did not convert into sinus rhythm. Importantly, Kaplan-Meier analysis of the survival curves for those who converted to SR with amiodarone as compared to those who did not showed significantly better survival [54]. Similar conclusions can be derived from DIAMOND trial [43] and the small CAFÉ-II study [55]. Again these are post hoc subgroup analyses and should be considered hypothesis generating rather than best available evidence. Kurita *et al. *argue in favour of rhythm control suggesting that overall prognosis in HF may improve provided without side effects of antiarrhythmic and catheter ablation complications [56]. 

A number of non-pharmacological modalities are in routine clinical use for the management of AF. Anti-arrhythmic drug therapy for the management of AF may in itself increase adverse cardiac events. AF-CHF trial patients who were in the rhythm control arm were more frequently hospitalized for dosage readjustment and cardioversion especially in the first year [42]. Furthermore, many patients are unable to achieve rhythm or rate control targets due to inadequacy of the drugs or side effects. Consequently, use of electrophysiological interventions to achieve this aim is increasing. AV nodal (AVN) ablation accompanied by a permanent pacemaker is often a last resort option for definitive AF rate control when medical therapy to achieve this has failed. This treatment strategy may only be of symptomatic benefit since AF is not eliminated and deleterious effects of A-V dysynchrony and loss of atrial transport still persist. While atrial lead placement and chronic atrial pacing has not shown any benefit in reducing AF recurrences, chronic RV pacing leads to progressive LV dysfunction due to inter-ventricular desynchronisation. As a result, upgrade to biventricular pacing has been suggested as a promising option provided it can be ensured that the device is pacing nearly 100% of the time for maximum benefit [57,58]. To date, a number of observational studies have shown improvement in LV function, reduction in mitral regurgitation and better exercise capacity with cardiac resynchronization therapy (CRT) in HF patients with AF [59–61]. For instance, the MUSTIC trial included 33 patients in AF, 29 (88%) of whom were programmed to biventricular pacing [59]. Similarly, registry data from Luedorff *et al. *looked at patients with severe heart failure incorporating 139 patients with AF vs. 445 in sinus rhythm. At 1 year follow up, CRT associated improvement of NYHA class and LV ejection fraction was similar in the two groups-albeit with higher mortality in AF group (12% vs. 7%; OR 1.80; 95% confidence interval 0.95-3.4) [61]. The patient numbers, however, are limited. Therefore, in patients requiring AVN ablation and a permanent pacemaker, CRT should be the pacing option of choice. Conversely, CRT is less effective if adequate rate control cannot be achieved in AF and in such cases AVN ablation can be very useful. AVERT-AF Trial [62] is currently underway to study the effect of AV junction ablation and CRT on patients with severely impaired LV systolic function and permanent AF looking at improvement in functional capacity. There is still a need for randomized, placebo-controlled trials looking at long term mortality data in patients with advanced heart failure.

Although AVN ablation and pacemaker implantation is an effective rate control strategy it does not eliminate the burden of AF. Moreover, biventricular pacing is potentially associated with a number of procedural risks. Consequently, catheter ablation (particularly pulmonary vein isolation) has gained popularity in the management of AF. One non-randomized trial of patients in AF studied catheter ablation results in 58 patients with LVEF < 45% compared to similar number of patients without HF. It showed a significant improvement in LVEF (mean increase of 21%) post ablation. There was also a significant improvement in symptoms, quality of life and exercise capacity (assessed by NYHA class, SF-36 quality of life scores and bicycle-ergometer stress test respectively). However, there was a high recurrence of AF and 50 % of the patients required a second procedure, although 79 % of patients remained in sinus rhythm at 1 year. The study was, however, under-powered to look at mortality trends [63]. A number of other small non–randomized studies have shown promising improvements in LVEF and patient symptoms [64–66]. PABA CHF looked at pulmonary vein isolation (PVI) vs. AV Node ablation plus biventricular ICD [67]. This was a prospective, multicentre, randomized trial enrolling drug-refractory AF patients with LVEF of 40% or less and in NYHA II/III. 41 patients underwent PVI while 40 had AVN ablation along with biventricular ICD implantation. The primary end point was a composite of LVEF, 6 minute walk distance and Minnesota Living with Heart Failure score. PVI patients did better in all three components of the composite end point than the group who underwent AVN ablation and biventricular pacing. This data suggests that perhaps optimal rate control with a regular RR length is not enough on its own and eliminating AF to restore atrial transport and AV synchrony is equally important [56]. Dagres *et al. *recently presented a meta-analysis of trials of catheter ablation for AF in patients with moderate LV systolic dysfunction. 9 studies incorporating a total of 354 patients were analysed. Primary end point was change in ejection fraction while secondary end points were changes in exercise tolerance and quality of life post procedure. Catheter ablation led to improvement in LV systolic function. However, the extent of this benefit was quite heterogeneous and no survival data is available [68]. Clearly, definitive large, multicentre, randomized controlled trials are needed with longer follow up to guide clinical practice. Surgical ablation (variations of Cox Maze procedure) is an effective option available to those who are undergoing cardiac surgery for other reasons [69]. It has been shown to be safe and effective in heart failure [70].

Heart failure patients with AF represent a cohort at very high risk of thromboembolic events. Long-term oral anticoagulation is strongly indicated in AF and HF unless there are binding contraindications. ACCF/AHA/HRS recommend either aspirin or anticoagulation for patients with a CHADS2 score of 1 while ESC and CCS guidelines indicate anticoagulation for such patients in preference to aspirin. There is unanimous recommendation of anticoagulation with a CHADS2 score of 2 and above [71]. Warfarin therapy is often underutilized due to a variety of limitations including erratic INR control, need for monitoring blood levels as well as interactions with various drugs/food. More recently, a novel group of anticoagulants has been developed with the advantage of rapid onset of action, predictable therapeutic levels not requiring monitoring as well as reduced risk of intracranial bleeding while maintaining efficacy. There is a low likelihood of interactions with drugs and food. There is, however, the caveat of higher cost, unavailability of antidote and no validated lab marker of anticoagulant effect when deemed clinically important [72]. The two main classes of these novel anti-coagulants include direct thrombin inhibitors (dabigatran) and activated factor X inhibitors (apixaban, rivaroxaban, edoxaban). Dabigatran was approved by FDA in 2010 for non-valvular AF following the Randomized Evaluation of Long-Term Anticoagulation Therapy (RE-LY) trial [73]. It showed that 150mg bd dose was superior in efficacy to warfarin while the lower 110 mg bd dose was at least non-inferior. Both had less risk of intracranial bleeding than warfarin. Dabigatran has been recommended as an alternative to warfarin in recent ESC as well as CCS guidelines as well [74,75]. ROCKET-AF (Rivaroxaban Once daily oral direct Factor Xa inhibition Compared with vitamin K antagonism for prevention of stroke and Embolism Trial in Atrial Fibrillation) studied once daily Rivaroxaban demonstrating non-inferiority to warfarin with reduced intracranial and similar rate of major bleeding [76]. ARISTOTLE (Apixaban^ ^for the Prevention of Stroke in Subjects With Atrial Fibrillation) trial demonstrated efficacy and safety of apixaban on similar grounds [77].

Finally, there has been a focus on modifying the arrythmogenic atrial substrate and neurohormonal milieu in order to prevent AF in heart failure patients. Limited data is available so far for statin therapy [78] and Renin-angiotensin-aldosterone system (RAAS) blockade [79,80]. In a recent meta-analysis of 8 RCTs incorporating 2323 patients, Bhuriya *et al. *looked at studies using ACE inhibitors or ARBs and containing data on outcomes of recurrent AF. They showed a significant reduction in recurrent AF in these patients (RR, 0.611; 95% CI, 0.441-0.847; P = .003). It should be pointed out, however, that the trials were not specifically designed to test this hypothesis and further large randomized controlled trials aimed a priori at the specific hypothesis are required [81].

## CONCLUSIONS

In conclusion, HF and AF frequently co-exist and the presence of AF in patients with HF has been reported to be independently associated with an increase in mortality in many studies and this increased risk is observed in those with both preserved and impaired LV systolic function. Whilst many studies have shown that the presence of AF in HF patients is associated with an adverse prognosis, most studies that have targeted AF in patients with HF with a view to maintaining SR have shown no significant improvements in outcomes compared to those patients in which a rate control strategy has been adopted. A number of small trials have studied the role of AF catheter ablation results in patients with HF and have shown modest improvement in LVEF as well as significant improvement in symptoms, quality of life and exercise capacity although the utility of this modality of treatment needs to be further investigated in larger randomised controlled trials. Finally, there has been a focus on modifying the arrythmogenic atrial substrate and neurohormonal milieu in order to prevent AF in heart failure patients although it remains to be seen whether this approach proves to be efficacious with improvements in clinically relevant outcomes.
